# Synthesis and optimization ring opening of monoepoxide linoleic acid using *p*-toluenesulfonic acid

**DOI:** 10.1186/2193-1801-2-429

**Published:** 2013-09-02

**Authors:** Jumat Salimon, Bashar Mudhaffar Abdullah, Rahimi M Yusop, Nadia Salih, Emad Yousif

**Affiliations:** School of Chemical Sciences & Food Technology, Faculty of Science and Technology, Universiti Kebangsaan Malaysia, 43600 Bangi Selangor, Malaysia

## Abstract

Biolubricant base oils, 9,12-hydroxy-10,13-oleioxy-12-octadecanoic acid (HYOOA) was synthesized based on the esterification reaction of Monoepoxide linoleic acid 9(12)-10(13)-monoepoxy 12(9)-octadecanoic acid (MEOA) with oleic acid (OA) and catalyzed by *p*-Toluenesulfonic acid. The optimum conditions for the experiment using D-optimal design to obtain high yield% of 84.61, conversion% of 83.54 and lowest OOC% of 0.05 were predicted at OA/MEOA ratio of 0.2:1 (mol/mol), PTSA/MEOA ratio of 0.4:1 (mol/mol), reaction temperature at 110°C, and reaction time at 4.5 h. The FTIR peaks of HYOOA indicate the disappearance of the absorption band at 820 cm^−1^, which belongs to the oxirane ring. ^13^C and ^1^H NMR spectra analyses confirmed the result of HYOOA with appearance carbon-ester (C = O) chemical shift at 174.1 ppm and at 4.06 ppm for ^13^C and ^1^H NMR respectively.

## Introduction

Plant oil based products have become more cost competitive with their petroleum derived counterparts as crude petroleum oil prices have increased dramatically in recent years due to a number of geopolitical factors (Moser et al. 2007). Fatty acid esters are natural oleochemicals and can be used for many industrial purposes. Oleic acid (OA) is one of the most important fatty acid. OA is generally considered being the predominant fatty acid in nature. Esters are one of the most important derivatives of OA. OA, esters of OA and various derivatives of OA can be used as a lubricating oil (Özgülsün & Karasmanoğlu 1999). A variety of chemical modifications of epoxidized plant oils and fatty acids are possible through epoxy moiety, and one of the most commonly used is the ring opening reaction. Due to the high reactivity of the oxirane ring, the epoxidation of the double bonds opens up a wide range of feasible reactions that can be carried out under moderate reaction conditions (Lozada et al. 2009).

Many studies have been carried out for oxirane ring opening using different alcohols, catalysts and carboxylic acids. The oxirane ring opening of epoxidized plant oils by hydrolysis and hydrogen peroxide in two-phase (polar-organic) systems, when a homogeneous acid catalyst is used was done by Campanella & Baltanás (2005). Acid hydrolysis of epoxidized plant oils is a very slow reaction that proceeds at the liquid-liquid inter phase. The results showed that the transport was controlled, as the influence of temperature was found to be almost negligible. While the degradation of epoxidized plant oils with hydrogen peroxide using an ion exchange resin (Amberlite IR-120) was also done by Campanella & Baltanás (2005). The result showed that the ring opening increased either by adding a higher amount of catalyst to the system or by decreasing the particle size of the catalyst since, in both cases, the external area of the catalyst became larger. The ring-opening reaction of epoxidized plant oil using Amberlyst 15 (Dry) catalysis was carried out by Lathi & Mattiasson (2007).

*p*-Toluenesulfonic acid (PTSA) was preferred to use in the reactions due by the maximum yield of epoxy ring opening and no evidence of any side reaction occurs during the reaction. A screening study was conducted to identify a catalyst that promotes epoxy ring opening of full epoxidized soybean oil avoiding side reactions at low concentration and temperature. Six catalysts different catalyst: formic acid, phosphoric acid, POLYCATVR 5, PTSA, POLYCATVR SA-1, and DABCOVR BL17 were evaluated in terms of acid number, oxirane oxygen content, and color analyses. PTSA shows a particular behavior that promotes the reaction resulting in a maximum oxirane oxygen content reduction; low acid number and color index compare to the others catalyst (Lozada et al. 2009).

Amongst these classes of products, hydroxyl esters find application as lubricants, polyurethane foams or casting resins. The physicochemical properties of lubricants derived from hydroxyl esters can be modified or altered using different carboxylic acids. The nature and size of the carbon chain, e.g., influence the product’s viscosity (Schuster et al. 2008).

In this study, the oxirane ring opening reaction of monoepoxide linoleic acid (MEOA) by the nucleophilic addition of OA in the presence of a homogeneous acid catalyst, such as *p*-toluenesulfonic acid (PTSA), to prepare 9,12-hydroxy-10,13-oleioxy-12-octadecanoic acid (HYOOA) is reported. The effects of different ratios of OA/MEOA, different ratios of PTSA/MEOA, different reaction temperatures, and different reaction times are analyzed by using D-optimal design. The experimental conditions and catalyst used were different from those previously used, leading to better selectivity towards the hydroxyl ester under mild working conditions.

## Results and discussion

### Reaction of synthesis of monoester

**Figure 1:**
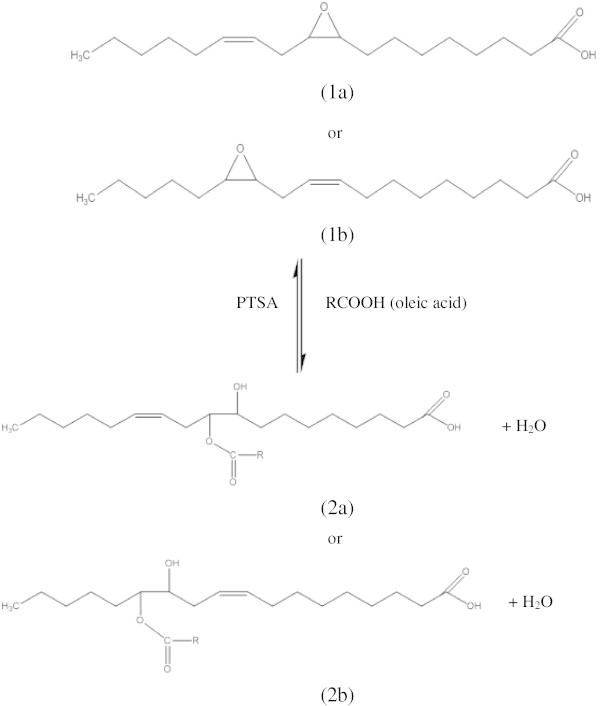
**Oxirane ring opening reaction to form HYOOA.** Notes: 9-10-monoepoxy 12-octadecanoic acid **(1a)**; 12-13-monoepoxy 9-octadecanoic acid **(1b)**; 9-hydroxy-10-oleioxy-12-octadecanoic acid **(2a)**; 12-hydroxy-13-oleioxy-9-octadecanoic acid **(2b)**; R=oleic acid.

Many nucleophilic reagents are known to add to an oxirane ring, resulting in ring opening (Salimon et al. 2012a). These ring opening reactions could result in branching at the oxirane ring opening (earlier sites of unsaturation in LA). The appropriate branching groups would interfere with the formation of macrocrystalline structures during low-temperature applications and would provide enhanced fluidity to plant oils. The ester branching groups produced from oxirane ring opening based esterification reaction are effective for attaining the desired molecular spacing. A chain length ester (saturated and unsaturated) has been observed to deliver the most desired physicochemical properties for some oils (Wu et al. 2008). These modified plant oils with chain branching are reported to have superior performance of the physicochemical properties and are promising as biolubricants (Hwang & Erhan 2001). This work reports the oxirane ring opening reaction of monoepoxide linoleic acid (MEOA) by the nucleophilic addition of oleic acid (OA) in the presence of homogeneous acid catalysts, such as *p*-toluene sulfonic acid (PTSA), to prepare 9,12-hydroxy-10,13-oleioxy-12-octadecanoic acid (HYOOA) (Figure 1) with yield% of 80.49.

### Effect of process parameters and statistical analysis

Optimization study of the oxirane ring-opening-based esterification reaction using D-optimal design took place in the presence of OA with using PTSA as a catalyst. The design is used to obtain 25 design points within the whole range of four factors for experiments. The designs and the response OOC% (*Y*) are given in Table 1 with measuring the yield% and IV mg/g. To see the impact on the oxirane ring opening by OA reaction, different ratios of OA/MEOA (mol/mol, *X*_1_), different ratios of PTSA/MEOA (mol/mol, *X*_2_), different reaction temperature (°C, *X*_3_), and different reaction time (h, *X*_4_) were evaluated.

**Table 1:** **D-optimal design arrangement and responses for HYOOA**

Run	Coded independent variable levels				Responses		
No.	OA^a^/MEOA^b^PTSA^c^/MEOA		Temp.	Time	OOC^d^	Yield	IV
	(mol/mol, X_1_)	(mol/mol, X_2_)	(°C, X_3_)	(h, X_4_)	(%, Y_1_)	(%, Y_2_)	(mg/g, Y_3_)
**Run**	**Coded independent variable levels**				**Responses**		
1	0.20	0.90	90	4.50	2.60	70.5	101.4
2	0.20	0.40	90	3.00	0.90	82.1	114.3
3	0.40	0.65	90	4.50	3.80	61.1	84.2
4	0.40	0.65	100	6.00	3.20	56.4	91.9
5	0.40	0.40	100	4.50	3.40	84.5	90.3
6	0.30	0.78	100	3.75	3.10	70.4	94.1
7	0.60	0.40	110	6.00	2.70	58.3	100.2
8	0.60	0.40	110	3.00	2.40	55.5	109.7
9	0.60	0.90	90	6.00	2.10	27.8	110.8
10	0.60	0.90	90	3.00	1.80	35.7	112.3
11	0.20	0.40	100	6.00	1.50	81.5	112.5
12	0.60	0.90	90	3.00	2.90	55.6	99.8
13	0.20	0.90	110	6.00	3.05	70.9	88.5
14	0.60	0.40	90	4.50	3.50	65.9	88.9
15	0.60	0.65	100	4.50	0.60	74.3	122.2
16	0.60	0.90	90	6.00	0.80	53.7	120.4
17	0.60	0.90	110	4.50	0.40	50.8	123.9
18	0.20	0.40	90	3.00	0.30	80.1	125.2
19	0.40	0.40	90	6.00	2.95	65.1	94.1
20	0.60	0.40	110	3.00	1.40	58.4	114.1
21	0.20	0.90	110	3.00	0.20	79.5	127.4
22	0.40	0.65	110	4.50	0.90	80.1	118.7
23	0.60	0.40	110	6.00	0.70	40.8	121.1
24	0.20	0.40	110	4.50	0.05	84.6	134.8
25	0.20	0.65	90	6.00	0.60	82.4	122.5

Notes: oleic acid (a); 9(12)-10(13)-monoepoxy 12(9)-octadecanoic acid (b); p-toluene sulfonic acid (c); oxirane oxygen content (d).

Table 1 illustrates the OOC%, yield% and IV mg/g effect related to the OA/MEOA ratio, PTSA/MEOA ratio, reaction temperature, and reaction time. As expected, at high temperature, 110°C, the OOC% shows a great reduction 0.05%, which was predicted at OA/MEOA ratio of 0.2:1 (mol/mol), PTSA/MEOA ratio of 0.4:1 (mol/mol) and reaction time at 4.5 h. This abrupt reduction on OOC% by high temperature (110°C) shows a high increment on yield, 84.61%, conversion, 83.54% and iodine value, 134.8 mg/g, compared with the initial iodine value (IV°) 77.65 mg/g. In light of these changes, other optimization experiments were discontinued.

As described in the mechanism, most of the oxirane ring groups are opened, and, consequently, were converted into ester bonds in the molecule with hydroxyl group. At lower temperatures, a fairly low oxirane ring reduction (3.80%) was observed at 90°C for 4.5 h of reaction compared with 0.05% at 110°C for 4.5 h. At 100°C, the OOC% shows a smooth reduction during the reaction Table 1.

The quadratic regression coefficients obtained by employing the least squares method to predict quadratic polynomial models for the OOC% of HYOOA (*Y*_*1*_), yield% (*Y*_*2*_) and IV mg/g (*Y*_*3*_) are given in Tables 2, 3 and 4. The OOC% response of HYOOA (*Y*_*1*_) shows that the linear term of reaction temperature (*X*_*3*_) was significant (*P* < 0.05) and highly significant (*P* < 0.01) for the IV mg/ g (*Y*_*3*_). The quadratic term of OA/MEOA ratio (*X*_11_) was highly significant (*P*<0.01) for both OOC% (*Y*_*1*_) and IV mg/g (*Y*_*3*_). The interaction terms of (*X*_*12*_) were highly significant (*P* < 0.01) for both OOC% (*Y*_*1*_) and IV mg/g (*Y*_*3*_), while the interaction terms of (*X*_*14*_) and (*X*_*34*_) were significant (*P*<0.05) for both OOC% (*Y*_*1*_) and IV mg/g (*Y*_*3*_). Highly significant (*P* < 0.01) terms of yield% (*Y*_*2*_) for the OA/MEOA (*X*_*1*_) were linear.

**Table 2:** **Regression coefficients of the predicted quadratic polynomial model for the response variables of the OOC% of HYOOA**

Variables	Coefficients (ß), OOC% (Y_1_)	***T***	***P***	Notability
Linear				
*X*_*1*_	0.23	1.18	0.3023	
*X*_*2*_	0.16	0.50	0.4970	
*X*_*3*_	-0.64	9.15	0.0128	**
*X*_*4*_	0.24	1.15	0.3088	
Quadratic				
*X*_*11*_	-1.92	13.10	0.0047	***
X_22_	1.17	4.75	0.0544	
X_33_	-0.56	1.31	0.2790	
X_44_	-2.386E-003	3.722E-005	0.9953	
Interaction				
X_12_	-0.91	14.95	0.0031	***
X_13_	-0.23	0.87	0.3725	
X_14_	-0.53	5.20	0.0457	**
X_23_	-0.072	0.095	0.7642	
X_24_	0.33	1.89	0.1988	
X_34_	0.54	5.14	0.0468	**
R^2^	0.81			

Notes: *X*_1_ = OA/MEOA ratio; *X*_2_ = PTSA/MEOA ratio; *X*_3_ = reaction temperature; *X*_4_ = reaction time. ** P < 0.05; *** *P* < 0.01. *T*: *F* test value See Table 1 for a description of the abbreviations.

**Table 3:** **Regression coefficients of the predicted quadratic polynomial model for the response variables of the yield% of HYOOA**

Variables	Coefficients (ß), yield% (Y_2_)	T	P	Notability
Linear				
*X*_*1*_	-11.76	18.38	0.0016	***
*X*_*2*_	-6.39	4.75	0.0542	
*X*_*3*_	1.11	0.17	0.6915	
*X*_*4*_	-2.57	0.81	0.3879	
Quadratic				
*X*_*11*_	6.50	0.91	0.3636	
*X*_*22*_	-5.68	0.67	0.4321	
*X*_*33*_	-4.02	0.41	0.5349	
*X*_*44*_	-8.11	2.58	0.1390	
Interaction				
*X*_*12*_	-0.068	5.008E-004	0.9826	
*X*_*13*_	-1.67		0.6037	
*X*_*14*_	-0.81	0.29	0.7922	
*X*_*23*_	2.97	0.073	0.3469	
*X*_*24*_	-0.64	0.97	0.8401	
*X*_*34*_	-1.81	0.043	0.5709	
*R*^*2*^	0.81	0.34		

Abbreviations: Notes: *X*_*1*_ OA/MEOA ratio, *X*_*2*_ PTSA/MEOA ratio, *X*_*3*_ reaction temperature, *X*_*4*_ reaction time. *** *P* < 0.01. *T*: *F* test value. See Table 1 for a description of the abbreviations.

**Table 4:** **Regression coefficients of the predicted quadratic polynomial model for the response variables of the IV mg/g of HYOOA**

Variables	Coefficients (ß), IV mg/g (***Y***_***3***_)	***T***	***P***	Notability
Linear				
*X*_*1*_	-2.79	1.54	0.2432	
*X*_*2*_	-2.02	0.71	0.4205	
*X*_*3*_	7.84	12.32	0.0056	***
*X*_*4*_	-3.17	1.85	0.2034	
Quadratic				
*X*_*11*_	22.75	16.54	0.0023	***
*X*_*22*_	-12.57	4.90	0.0513	
*X*_*33*_	6.80	1.76	0.2144	
*X*_*44*_	-0.30	5.153E-003	0.9442	
Interaction				
*X*_*12*_	10.60	18.35	0.0016	***
*X*_*13*_	1.76	0.47	0.5064	
*X*_*14*_	5.52	5.06	0.0482	**
*X*_*23*_	-1.24	0.25	0.6247	
*X*_*24*_	-3.79	2.23	0.1663	
*X*_*34*_	-6.97	7.61	0.0202	**
*R*^*2*^	0.84			

Notes: *X*_1_ = OA/MEOA ratio; *X*_2_ = PTSA/MEOA ratio; *X*_3_ = reaction temperature; *X*_4_ = reaction time. ** *P* < 0.05; *** *P* < 0.01. *T*: *F* test value. See Table 1 for a description of the abbreviations.

From the experimental results in Table 1 and Equations (1, 2, 3) a second order polynomial equation was developed (in coded units) that could relate the OOC% (*Y*_*1*_), yield% (*Y*_*2*_) and IV mg/g (*Y*_*3*_) of HYOOA to the parameters under study. The following quadratic model is explained in Equations (1, 2 and 3).123

The lack of fit *F*-value for the all responses *Y*_*1*_, *Y*_*2*_ and *Y*_*3*_ shows that the lack of fit is not significant (*P*>0.05) relative to the pure error. This indicates that all the models predicted for the responses were adequate. The regression models for the data on responses *Y*_*1*_, *Y*_*2*_ and *Y*_*3*_ were significant (*P*<0.05) with satisfactory *R*^2^. However, the *R*^2^ for the *Y*_*1*_, *Y*_*2*_ and *Y*_*3*_ were (0.81, 0.81 and 0.84, respectively), although the model was significant. Table 5 summarizes the analysis of variance (ANOVA) of all the responses *Y*_*1*_, *Y*_*2*_ and *Y*_*3*_ of this study.

**Table 5:** **Analysis of variance (ANOVA) for all the responses of HYOOA**

	Source	***Df***	Sum of squares	Mean square	***F*** value	***Prob*** > ***F***	
	Model	14	30.35	2.17	3.19	0.0357	Significant
*Y*_*1*_	Residual	10	6.80	0.68			
	Lack-of-fit	5	2.67	0.53	0.65	0.6783	Not significant
	Pure error	5	4.13	0.83			
	Model	14	4862.62	347.33	3.07	0.0402	Significant
*Y*_*2*_	Residual	10	1130.50	113.05			
	Lack-of-fit	5	437.76	87.55	0.63	0.6866	Not significant
	Pure error	5	692.74	138.55			
	Model	14	4283.91	305.99	4.03	0.0161	Significant
*Y*_*3*_	Residual	10	758.84	75.88			
	Lack-of-fit	5	347.14	69.43	0.84	0.5719	Not significant
	Pure error	5	411.69	82.34			

The significant interaction variables in the fitted models (Tables 2, 3 and 4) were chosen as the axes (OA/MEOA ratio; *X*_1_, PTSA/MEOA ratio; *X*_2_, reaction temperature; *X*_3_ and reaction time; *X*_4_) for the response surface plots. The relationships between independent and dependent variables are shown in the three-dimensional representation as response surfaces. In a contour plot, the curves of equal response values are drawn on a plane whose coordinates represent the levels of the independent factors. Each contour represents a specific value for the height of the surface above the plane defined for combination of the levels of the factors. Therefore, different surface height values enable one to focus attention on the levels of the factors at which changes in the surface height occur (Hwang & Erhan 2001).

**Figure 2:**
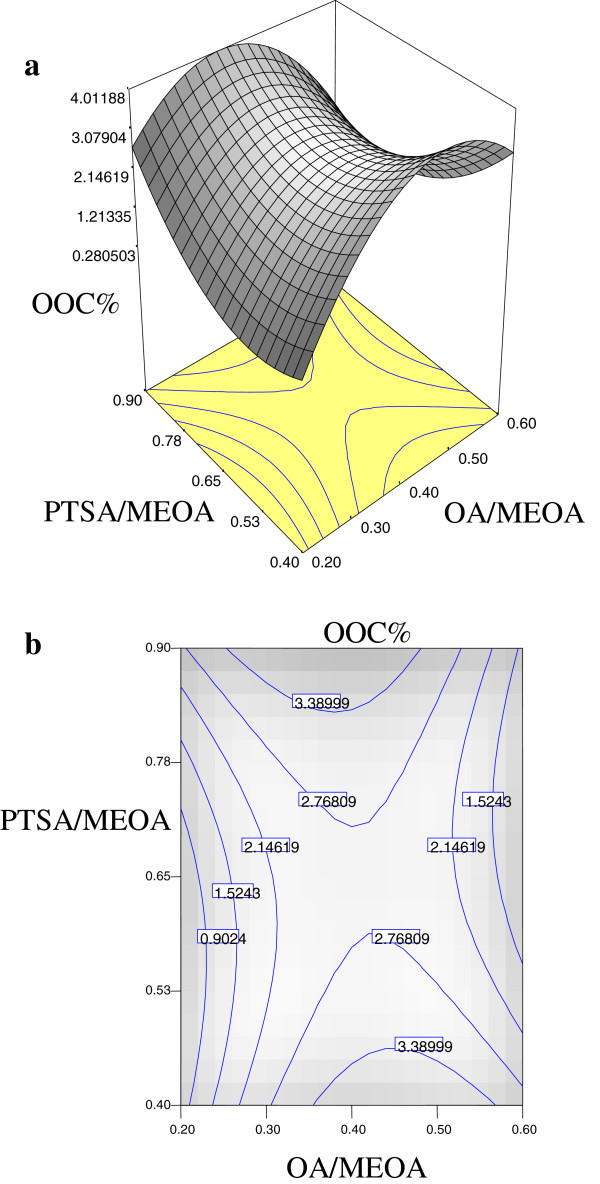
**Response surface (a) and contour plots (b) for the OOC%.** Response surface **(a)** and contour plots **(b)** for the OA/MEOA ratio (X1,mol/mol) and PTSA/MEOA ratio (X2, mol/mol) on the OOC%.

**Figure 3:**
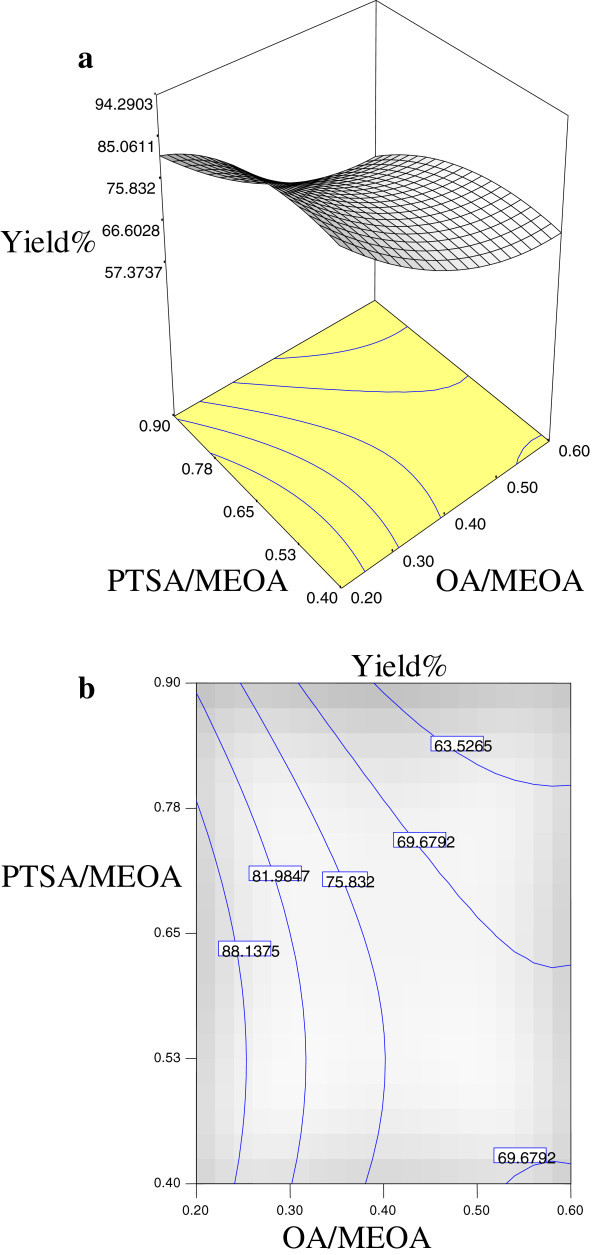
**Response surface (a) and contour plots (b) for the yield%.** Response surface **(a)** and contour plots **(b)** for the OA/MEOA ratio (X1,mol/mol) and PTSA/MEOA ratio (X2, mol/mol) on the yield%.

**Figure 4:**
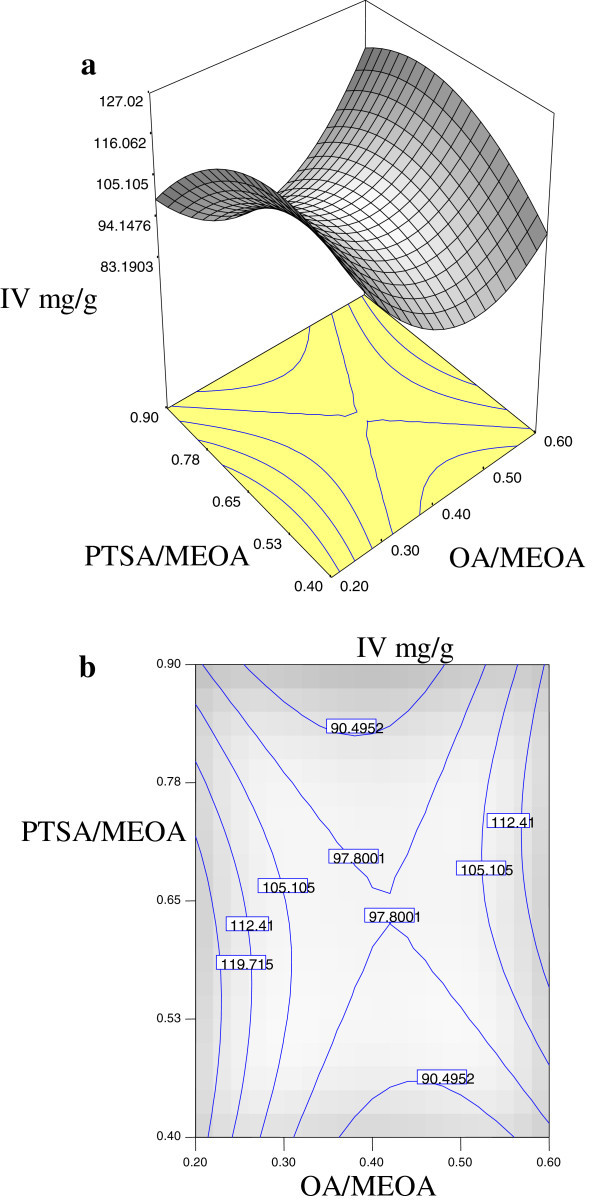
**Response surface (a) and contour plots (b) for the IV mg/g.** Response surface **(a)** and contour plots **(b)** for the OA/MEOA ratio (X1,mol/mol) and PTSA/MEOA ratio (X2, mol/mol) on the IV mg/g.

**Figure 5:**
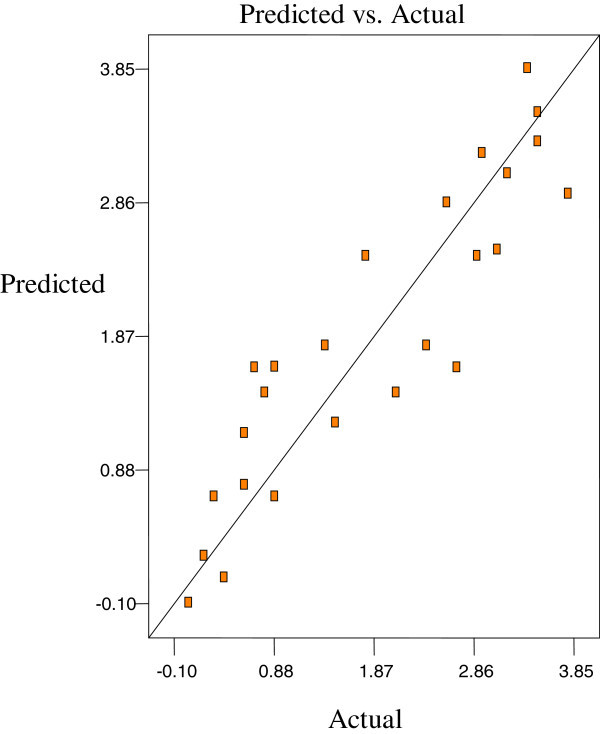
**Predicated vs. actual plot of Y1.**

**Figure 6:**
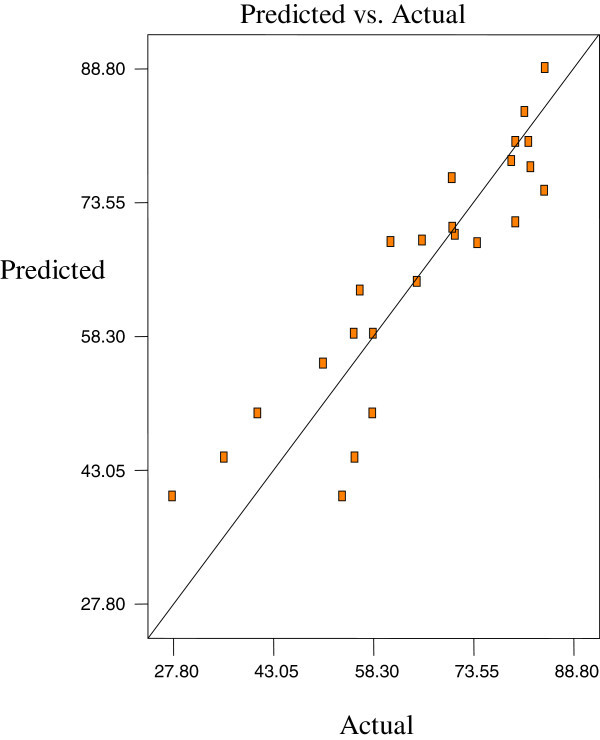
**Predicated vs. actual plot of Y2.**

**Figure 7:**
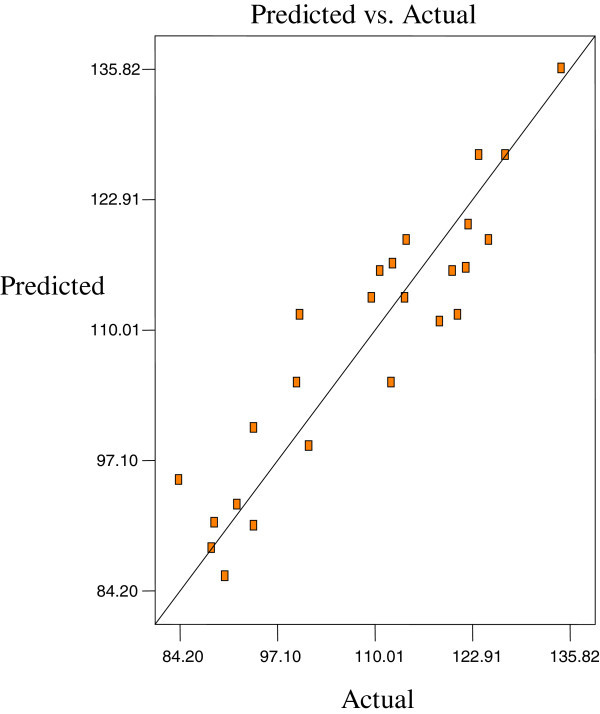
**Predicated vs. actual plot of Y3.**

Figures 2, 3 and 4 are the Design-Expert plots for all the responses. In the oxirane ring opening reaction for HYOOA, performing the technique using high reaction temperature would give the desired OOC% of HYOOA. The relationships between the parameters and oxirane ring opening of HYOOA were linear or almost linear. Reconfirmation of the optimal condition experiment was repeated three times. The observed value was reasonably close to the predicted value, as shown in Figures 5, 6 and 7.

The lowest OOC of 0.05% could be obtained by using a low ratio of OA/MEOA and low ratio of PTSA/MEOA at high reaction temperature, such as other studies, which have used a lower PTSA ratio (Salimon et al. 2012a). Experimental variables should be carefully controlled in order to reduce the OOC% of interest with reasonable yield%. Optimum conditions of the experiment to obtain high yield% of HYOOA and lowest OOC% were predicted at a ratio of OA/MEOA of 0.2:1 (mol/mol), ratio of PTSA/MEOA of 0.4:1 (mol/mol), reaction temperature 110°C, and 4.5 h reaction time. In this condition, the OOC of HYOOA was 0.05%, yield was 84.61%, and IV was 134.8 mg/g (Table 1).

### FTIR analysis of HYOOA

**Figure 8:**
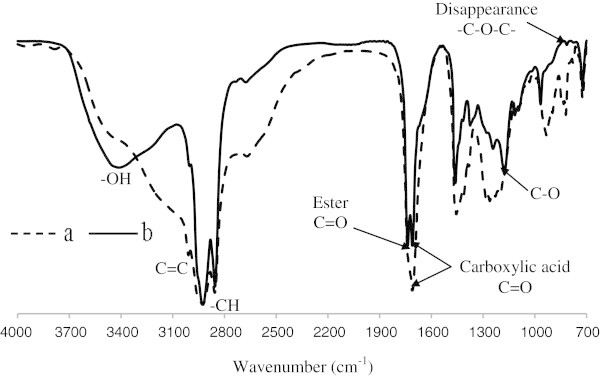
**FTIR spectra of the MEOA (a) and HYOOA (b).**

The spectrum from the FTIR analysis displays several absorption peaks, as shown in Figure 8. The main peaks and their assignment to functional groups are given in Table 6. The FTIR peaks of HYOOA indicate the disappearance of the absorption band at 820 cm^−1^, which belongs to the oxirane ring. For the ester carbonyl functional groups C = O of HYOOA at 1741 cm^−1^ and the carboxylic carbonyl vibration functional group at 1711 cm^−1^, which show the same absorption band in MEOA at 1711 cm^−1^. The FTIR peaks at 2925 to 2855 cm^−1^ indicate the CH_2_ and CH_3_ scissoring of MEOA and HYOOA.

**Table 6:** **The main wavenumbers in the FTIR functional groups of MEOA and HYOOA**

Wavenumber of MEOA^a^	Wavenumber of HYOOA^b^	Functional group
-	3413	OH stretching (alcohol)
-	1737	C = O stretching vibration (ester)
-	1176, 1117	C-O bending vibration (ester)
820	-	C-O-C oxirane ring

Notes: Monoepoxide linoleic acid (a); 9,12-hydroxy-10,13-oleioxy-12,9-octadecanoic acid (b).

The FTIR spectroscopy analysis of MEOA and HYOOA indicate the presence of a peak at 3003-3008 cm^−1^, which belongs to the double bond C = C (stretching aliphatic), while at 3413 cm^−1^, it belongs to OH stretching of HYOOA. The peaks at 1176 and 1117 cm^−1^of HYOOA are referred to as (C–O) stretching ester. FTIR spectrum also shows absorption bands at 723 cm^−1^ for (C–H) group vibration. A similar observation has been reported for the FTIR spectrum of oxirane ring opening of epoxide oleic acid (Salimon et al. 2012a).

### ^1^H And ^13^C NMR analysis of HYOOA

#### a. ^1^H NMR analysis

**Figure 9:**
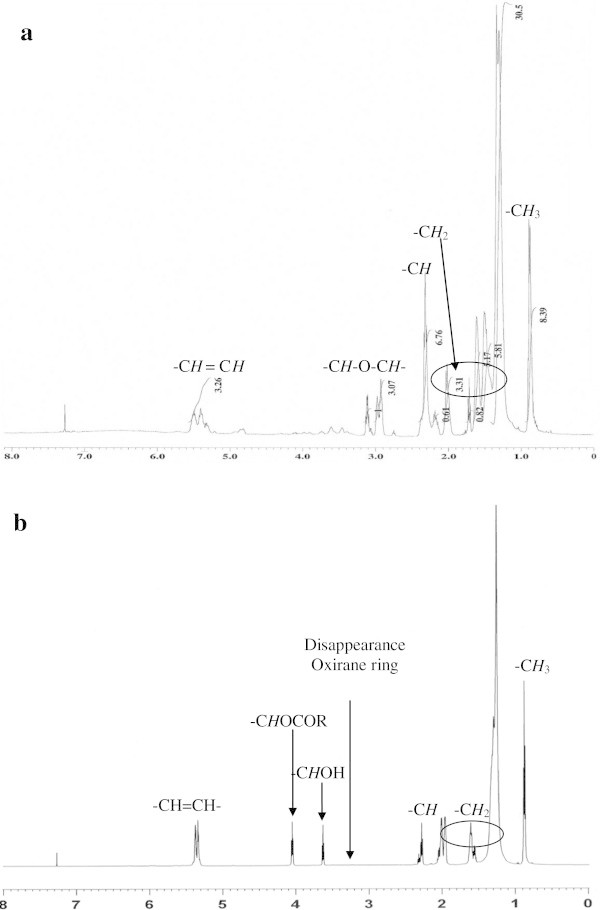
**1H NMR spectrum of MEOA (a) and HYOOA (b).**

^1^H NMR spectroscopy shows the main signals assignments of MEOA and HYOOA, as shown in Table 7. The ^1^H NMR spectra for the products HYOOA show the disappearance of the ring opening (-CH-O-CH-) in the range of 2.92-3.12 ppm, which refers to the MEOA, Figure 9. The distinguishable peaks appear in HYOOA -CH-OH at 3.62 ppm while -CHOCOR at 4.06 ppm, which did not appear in MEOA (Figure 9a and b).

**Table 7:** **The main signals present in**^**1**^**H NMR functional groups of MEOA and HYOOA**

δ (ppm) of MEOA^a^	δ (ppm) of HYOOA^b^	Assignment
0.86–0.88	0.82–0.84	-C*H*_3_
1.29–2.03	1.23–2.06	-C*H*_2_
2.29–2.33	2.26–2.33	-C*H*
2.92–3.12	-	-C*H*-O-C*H*-
-	3.62	-C*H*OH
-	4.06	-C*H*OCOR
5.38–5.49	5.31–5.40	-C*H* = C*H*-

Notes: Mono-epoxide linoleic acid (a); 9,12-hydroxy-10,13-oleioxy-12,9-octadecanoic acid (b).

The signals at 0.82-0.84 ppm refer to the methylene group (-CH_3_) of HYOOA, which also appears in MEOA next to the terminal methyl (-CH_2_) at 1.23-2.06 ppm of HYOOA. The other distinctive signals were methine at about 2.26-2.33 ppm, which are common for these types of compounds (Doll et al. 2007). However, the methane proton signal (-CH = CH-) was shifted upfield to about 5.31-5.40 ppm of HYOOA.

### b. ^13^C NMR analysis

**Figure 10:**
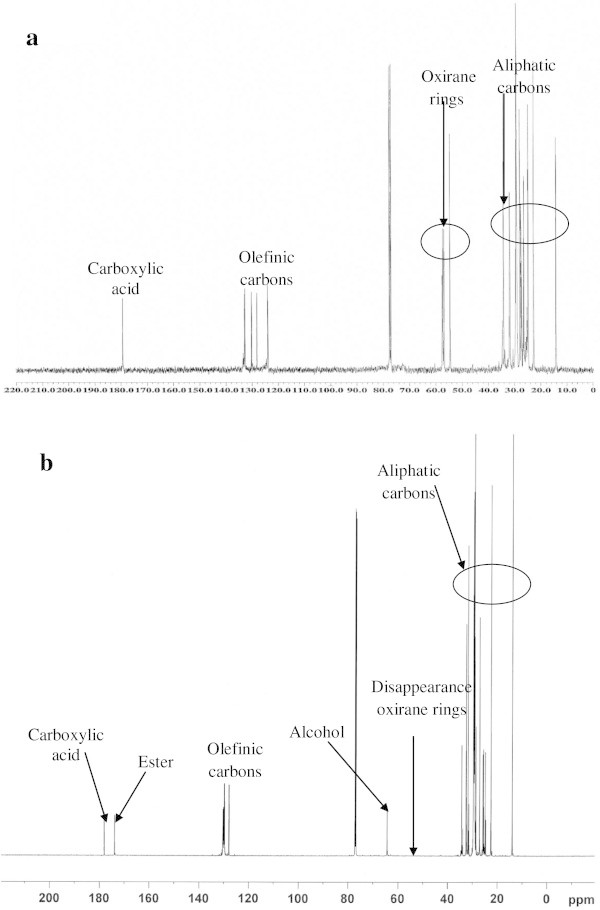
**13C NMR spectrum of MEOA (a) and HYOOA (b).**

Figures 10a and b indicate the ^13^C NMR spectrum of MEOA and HYOOA, respectively. The ^13^C spectroscopy shows the main signals assignment of MEOA and HYOOA, as shown in Table 8. The signals at 179.32 and 178.11 ppm refer to the carbon atom of the carbonyl group (carboxylic acid) for MEOA and HYOOA, respectively, while 174.1 ppm appear in HYOOA, which refers to the ester group. The signals at 127.90 to 130.57 ppm refer to the unsaturated carbon atoms (olefin carbons) for both MEOA and HYOOA.

**Table 8:** **The main signals present in**^**13**^**C NMR functional groups of MEOA and HYOOA**

δ (ppm) of MEOA^a^	δ (ppm) of HYOOA^b^	Assignment
22.69–34.15	25.76–34.38	Aliphatic carbons
54.59–57.29	-	() Epoxide groups
-	64.41	-OH alcohol
124.02–132.89	127.90–130.57	-CH = CH- Olefinic carbons
-	174.01	C = O Ester
179.32	178.11	C = O Carboxylic acid

Notes: Mono-epoxide linoleic acid (a); 9,12-hydroxy-10,13-oleioxy-12,9-octadecanoic acid (b).

Figure 10b confirms the disappearance of the oxirane ring, which appeared in MEOA at 54.59-57.29 ppm and the appearance OH alcohol in HYOOA at about 64.41 ppm. The other distinctive signals were aliphatic carbons HYOOA at about 25.76-34.38 ppm, which are common for these types of compounds, and belong to the methylene carbon atoms of MEOA and HYOOA (Salimon et al. 2012a).

### Methodology

#### Experimental procedure

The oxirane ring opening reaction was carried out using oleic acid (OA) and p-toluene sulfonic acid (PTSA) as catalyst to prepare 9,12-hydroxy-10,13-oleioxy-12-octadecanoic acid (HYOOA) (Salimon et al. 2012a). Table 9 shows the different OA/MEOA ratios, different PTSA/MEOA ratios, different reaction temperatures and different reaction times using D-Optimal design. Factors such as ratio OA/MEOA (mol/mol, X_1_), PTSA/MEOA (mol/mol, X_2_), reaction temperature (°C, X_3_) and reaction time (h, X_4_) were performed under the same experimental conditions. MEOA (1.55 g; 0.005 mol) and ratio PTSA/MEOA (0.2:1-0.6:1 mol/mol) were dissolved in toluene (10 mL) in a 250-mL three-neck flask equipped with a cooler, dropping funnel and thermometer. The mixture was kept at 50°C. OA/MEOA ratio (0.4:1-0.9:1 mol/mol) was added during 1.5 h in order to keep the reaction mixture temperature under 70-80°C. The reaction mixture was subsequently heated to different temperatures 90-110°C, and refluxed at different times 3-6 h, at this temperature range. After reaction termination, the heating was stopped and the mixture was left to stand overnight at ambient temperature. The mixture was washed with the water and the organic layer was dried over anhydrous sodium sulphate and the solvent was removed using the vacuum evaporator. The oxirane ring content (OOC%), yield% and iodine value (IV mg/g) were measured. The FTIR, ^1^H, ^13^C were analyzed.

**Table 9:** **Independent variables and their levels for D-optimal design of the oxirane ring opening reaction**

Independent variables		Variable levels		
		-1	0	+1
1. OA/MEOA (mol/mol)	*X*_*1*_	0.2	0.4	0.6
2. PTSA/MEOA (mol/mol)	*X*_*2*_	0.4	0.65	0.9
3. Temperature (°C)	*X*_*3*_	90	100	110
4. Time (h)	*X*_*4*_	3	4.5	6

### Experimental design and statistical analysis

To explore the effect of the operation variables on the response in the region of investigation, a D-optimal design was performed. Ratio of OA/MEOA (mol/mol, *X*_1_), ratio of PTSA/MEOA (mol/mol, *X*_2_), reaction temperature (°C, *X*_3_), and reaction time (h, *X*_4_) were selected as independent variables. The range of values and coded levels of the variables are given in Table 9. Each variable to be optimized was coded at three levels: -1, 0, and +1. A quadratic polynomial regression model was assumed for predicting individual Y variables. The model proposed for each response of Y was:4

Where ß_0_; ß_i_; ß_ii_ and ß_ij_ are constant, linear, square and interaction regression coefficient terms, respectively, and *x*_*i*_ and x_j_ are independent variables. The Minitab software version 14 (Minitab Inc., USA) was used for multiple regression analysis, analysis of variance (ANOVA), and analysis of ridge maximum of data in the response surface regression (RSREG) procedure. The goodness of fit of the model was evaluated by the coefficient of determination R^2^ and the analysis of variance (ANOVA). Response surfaces and contour plots were developed using the fitted quadratic polynomial equations obtained from RSREG analysis and holding the independent variables with the least effect on the response at two constant values and changing the levels of the other two variables (Salimon et al. 2012b).

## Conclusion

In this work described the process of a systematic approach to chemically modify the MEOA to yield monoester (HYOOA) biolubricant capable of operating at low temperatures. The optimum conditions for the experiment using D-optimal design to obtain high yield% of 84.61, conversion% of 83.54 and lowest OOC% of 0.05 were predicted at OA/MEOA ratio of 0.2:1 (mol/mol), PTSA/MEOA ratio of 0.4:1 (mol/mol), reaction temperature at 110°C, and reaction time at 4.5 h.
